# Circulating tumor DNA in Hodgkin lymphoma

**DOI:** 10.1007/s00277-022-04949-x

**Published:** 2022-09-08

**Authors:** Maria Maco, Kristyna Kupcova, Vaclav Herman, Iva Ondeckova, Tomas Kozak, Heidi Mocikova, Ondrej Havranek, On behalf of the Czech Hodgkin Lymphoma Study Group

**Affiliations:** 1 Department of Hematology, University Hospital Kralovske Vinohrady, Third Faculty of Medicine, Charles University, Prague, Czech Republic; 2 BIOCEV, First Faculty of Medicine, Charles University, Prumyslova 595, 25250 Vestec, Czech Republic; 3 First Department of Medicine - Hematology, General University Hospital, Prague, Czech Republic

**Keywords:** Hodgkin lymphoma, Risk factors, Circulating tumor DNA, Genomic profile, ctDNA

## Abstract

Somatic mutations of genes involved in NF-κB, PI3K/AKT, NOTCH, and JAK/STAT signaling pathways play an important role in the pathogenesis of Hodgkin lymphoma (HL). HL tumor cells form only about 5% of the tumor mass; however, it was shown that HL tumor-derived DNA could be detected in the bloodstream. This circulating tumor DNA (ctDNA) reflects the genetic profile of HL tumor cells and can be used for qualitative and quantitative analysis of tumor-specific somatic DNA mutations within the concept of liquid biopsy. Overall, the most frequently mutated gene in HL is *STAT6*; however, the exact spectrum of mutations differs between individual HL histological subtypes. Importantly, reduction of ctDNA plasma levels after initial treatment is highly correlated with prognosis. Therefore, ctDNA shows great promise as a novel tool for non-invasive tumor genome analysis for biomarker driven therapy as well as for superior minimal residual disease monitoring and treatment resistance detection. Here, we summarize the recent advancements of ctDNA analysis in HL with focus on ctDNA detection methodologies, genetic profiling of HL and its clonal evolution, and the emerging prognostic value of ctDNA.

## Introduction

Hodgkin lymphoma (HL) is a very specific cancer type. In contrast to other cancers, HL malignant tumor cells (the Hodgkin and Reed-Sternberg, HRS cells) form minority of the cellular infiltrate within the HL tumors. According to the World Health Organization (WHO) classification of tumors of hematopoietic and lymphoid tissues, frequency of HRS cells can range from 0.1 to 10% [1]. In majority of HL tumors, the HRS cells frequency is around 1% [2, 3]. The remaining part consists of tumor infiltrating immune cells which form HL tumor’s specific inflammatory microenvironment [4]. HRS cells are derived from germinal center B cells but lack expression of surface antigens typical for B cells (CD19 and CD20). Characteristic surface antigens of HRS cells are CD30 and CD15 [4]. Though the annual incidence of HL is relatively low, approximately 2–3 new cases per 100,000 inhabitants per year in western countries, it is one of the most frequent cancer types in young adults [5, 6]. A majority of HL patients (80–90%) can be cured with conventional chemoradiotherapy [7–9]. However, 10–20% of patients younger than 60 years and almost 50% of patients older than 60 years relapse after first line treatment and relapse of HL is generally associated with unfavorable prognosis [10–15].

Multiple systems of predictive factors and scores were developed for patient stratification and therapy outcome prediction to guide the selection of the most appropriate type and intensity of chemotherapy and radiotherapy (RT). Currently, the most commonly used HL negative prognostic factors are high erythrocyte sedimentation rate (ESR), massive mediastinal tumor, extranodal involvement, and involvement of 3 or more lymph node areas for early stage HL patients (risk factors slightly differ between study groups, Table 1) and the international prognostic score (IPS) for advanced stage patients [16, 17]. Staging of HL is based on modified Ann-Arbor classification. Patients in clinical stage I–II without risk factors are defined as early stage favorable. Patients in clinical stage I–II with at least one risk factor are defined as early stage unfavorable. Advanced disease is defined as a clinical stage III or IV. According to the German Hodgkin Study Group (GHSG), patients in clinical stage IA, IB, or IIA with at least one risk factor and patients in clinical stage IIB with high ESR and/or ≥ 3 involved lymph node areas are defined as intermediate stage and patients with clinical stage IIB and a large mediastinal mass or extranodal disease are classified as advanced stage. The IPS for advanced stage HL defines seven adverse prognostic factors: serum albumin < 4 g/dL, hemoglobin < 10.5 g/dL, male sex, age ≥ 45 years, stage IV disease, white cell count ≥ 15,000/mm^3^, and lymphocyte count < 600/mm^3^ (or lymphocyte count < 8% of white cell count). On the other hand, recent studies suggested that IPS becomes much less discriminative with current standard therapy and PET-adapted treatment (see below) [18, 19]. Unfortunately, none of the above-mentioned prognostic factors accurately identifies patients who will respond poorly to the initial treatment, including initial positron emission tomography/computed tomography (PET/CT)-based assessment of the total metabolic tumor volume. Therefore, our ability to accurately differentiate patients who are at high risk of relapse and might require more intensive treatment is limited.

**Table 1:** Comparison of unfavorable risk factors for stage I–II Hodgkin lymphoma

Risk factor	GHSG [17]	EORTC [20]
Erythrocyte sedimentation rate	≥ 50 (A) or ≥ 30 (B)	≥ 50 (A) or ≥ 30 (B)
Massive mediastinal tumor	Mediastinal thoracic ratio > 1/3	Mediastinal thoracic ratio ≥ 0.35
Extranodal involvement	Yes	No
Nodal areas	≥ 3 areas	≥ 4 areas
Age	No	≥ 50 years

Abbreviations: *A*, without B symptoms; *B*, with B symptoms; *GHSG*, German Hodgkin Study Group; *EORTC*, European Organization for Research and Treatment of Cancer

Fluorodeoxyglucose (FDG) PET/CT is currently the standard imaging method for initial staging as well as for interim and final assessment of chemotherapy and chemoimmunotherapy outcome in lymphomas including HL. The Lugano classification uses a 5-point scale for PET/CT assessment of interim and final response [21]. The lymphoma response to immunomodulatory therapy criteria (LYRIC) further refines response to immunomodulatory agents, such as checkpoint inhibitors [22]. Interim PET/CT after two cycles of chemotherapy is a stronger predictor of outcome than any pretreatment prognostic factor; however, the exact positive and negative prognostic values in early and advanced stages are still under debate [23–26]. PET-adapted treatment strategies in early stages of HL tried to omit RT to reduce treatment-related toxicity. PET negativity after two or three cycles of ABVD (doxorubicin, bleomycin, vinblastine, and dacarbazine) resulted in increased risk of relapse when RT was omitted [9, 27, 28]. Based on the GHSG HD16 trial, the current standard of treatment for early stage favorable HL consists of two cycles of ABVD and involved site (IS) RT with 20 Gy [9]. The EORTC/LYSA/FIL H10 trial for early favorable and unfavorable stages of HL showed good result of treatment adaptation after two cycles of ABVD: PET-2 positive patients were switched to two cycles of BEACOPPesc (bleomycin, etoposide, doxorubicin, cyclophosphamide, vincristine, procarbazine, and prednisone) and involved-node RT (INRT); PET-2 negative patients continued with ABVD and INRT [29]. The GHSG HD17 trial for early unfavorable stages of HL showed that patients who are PET negative after two cycles of BEACOPPesc and two cycles of ABVD do not require consolidation with INRT and PET-4 positive patients should continue with INRT of 30 Gy [7]. For advanced stage HL, studies reported that PET-2 may help to identify patients in need of more intensive treatment or allow treatment de-escalation. According to the GHSG HD18 trial, PET-2 negative patients after two cycles of BEACOPPesc can finish the treatment with two additional cycles of BEACOPPesc. The PET-2 positive patients should continue with four additional cycles of BEACOPPesc [30]. Consolidation RT is indicated in PET-6 positive cases [31]. As an example of treatment de-escalation, the LYSA AHL2011 trial indicated that PET-2 negative patients after two cycles of BEACOPPesc can by switched to ABVD [8]. According to the RATHL trial, the omission of bleomycin from ABVD in PET-2 negative patients lowered incidence of pulmonary toxicity without affecting treatment efficacy. PET-2 positive patients after two cycles of ABVD were treated with BEACOPPesc [32]. The exact prognostic value of interim PET/CT in HL is still subject of further evaluation and HL patients will probably benefit from inclusion of additional biomarkers to complement the PET-adaptive treatment strategies.

From a large number of novel biomarkers, one of the most promising non-invasive approaches for HL patients risk stratification is based on analysis of circulating tumor DNA (ctDNA). In this review, we summarize the status and latest advances of ctDNA analysis in HL.

## Cell free DNA, circulating tumor DNA, and methodological issues

It has been shown that non-cellular DNA can be detected in various body compartments and/or fluids. This non-cellular DNA could be present in extracellular vesicles (e.g., exosomes) or freely in each compartment and/or fluid. Although there are studies of exosomal DNA in cancer, most studies of non-cellular DNA focus on non-vesical DNA [33]. This non-cellular non-vesical DNA, termed cell-free DNA (cfDNA), is normally present in all healthy individuals and is generated during multiple normal cellular processes including apoptosis, necrosis, or active secretion of extracellular vesicles and exosomes. One of the main sources of cfDNA are hematopoietic cells [34]. cfDNA was thought to be in majority a double stranded and naturally fragmented DNA (around 170 base pairs long) usually present at low concentrations; e.g., about 5 ng (rarely more than 30 ng) of cfDNA per ml of plasma in healthy individuals [35]. Interestingly, recent studies suggested that a substantial proportion of cfDNA might consists of single stranded “ultrashort” cfDNA (centered around 50 base pairs in length); however, specific approach is necessary for single strand cfDNA extraction and analysis [36, 37]. ctDNA represents the fraction of circulating cfDNA that originated from tumor cells. The capability to detect and analyze this tumor-specific DNA from plasma, serum, or other body fluids led to the concept of a “liquid biopsy” [38]. In comparison to the “classic” tumor biopsy, the main advantages of ctDNA analysis are minimal invasivity, reflection of the tumor heterogeneity across all affected tumor sites, and easy repetitive sampling allowing longitudinal assessment in various timepoints.

Depending on the stage and location of tumor, ctDNA can be found in a variety of body fluids, including blood plasma, urine, sputum, cerebrospinal fluid, pleural fluid, and saliva [39–44], but low levels of ctDNA may represent a substantial issue for its analysis. The amount of required input cfDNA is dependent on the analysis technique and approach. For analysis from plasma, the required minimal amount of blood to extract cfDNA usually ranges between 6 and 10 ml [45]. Several studies have indicated that serum has substantially higher concentrations of cfDNA compared to plasma, but this is mainly due to the aggregation and consequent lysis of leukocytes, which therefore contaminates cfDNA with leukocytic genomic DNA. In contrast to plasma, the degree of genomic DNA contamination in serum is significantly dependent on the storage temperature of samples and the time between blood collection and processing. As genomic DNA contamination of the extracted cfDNA significantly affects the ability to accurately analyze the ctDNA, the current consensus is to use plasma for cfDNA extraction [39, 46, 47]. Moreover, comparison of methodological procedures of cfDNA extraction did not show any significant effect of plasma storage temperature (room temperature or 4 °C) on cfDNA yield [48]. Based on the minimal period of cfDNA stability reported in several studies, blood collected in a tube with EDTA should ideally be processed within 6 h of venipuncture, though this is not strictly necessary with special cell free DNA blood collection tubes containing leukocyte lysis blocking reagents that allow storage of collected blood samples at room temperature for several days before processing. After centrifugation, plasma samples can be stored at − 80 °C until later cfDNA extraction [45]. In comparison to the ctDNA analysis from plasma, the detection of ctDNA in non-blood fluids such as urine, sputum, saliva, cerebrospinal fluid, or pleural fluid is much less studied. The benefits of ctDNA analysis in non-blood compartments include completely non-invasive collection for fluids like urine or saliva and, more importantly, allows analysis of ctDNA in a compartment anatomically close to the tumor site. Non-blood compartments were analyzed mainly in solid tumors, e.g., urinary tract cancers, colorectal cancer, non-small cell lung cancer, or tumors of the central nervous system [41–44, 49]. Mutter et al. recently demonstrated feasibility of cerebrospinal fluid ctDNA analysis for longitudinal disease burden monitoring in patients with central nervous system lymphomas [50].

## Circulating tumor DNA detection methods

Cancer is generally considered a genetic oligoclonal disease. During its development and progression, the tumor cells acquire multiple driver as well as passenger somatic DNA mutations [51]. Detection of these acquired tumor specific DNA mutations within cfDNA is the basis of all methods of ctDNA analysis. There are two general approaches: (1) detection of one or a small number of cancer type and/or tumor specific hot-spot mutations (e.g., by digital droplet PCR — ddPCR or classic real time PCR) and (2) next generation sequencing (NGS)-based methods usually assessing panel of selected cancer specific genes.

The digital PCR (dPCR) methods are modifications of classical PCR that can be used for both qualitative and quantitative analysis of DNA. The most used dPCR is the ddPCR [52]. The combination of ddPCR with allele specific PCR was used to detect and quantify tumor-specific mutations (e.g., the *STAT6* N417Y mutation in HL) [53], gene fusions (e.g., the *KIAA1549:BRAF* fusion in glioma) [54], or gene rearrangements (e.g., the *BCL2/IGH* in follicular lymphoma or the *EML4/ALK* in lung adenocarcinoma) [55, 56].

However, NGS-based methods have been used in the majority of recent ctDNA studies due to recent advances in technology, superiority in the amount of information that can be learned about the tumor, and wider availability. In lymphomas, generally two NGS-based approaches can be used: immunoglobulin genes sequencing (IgNGS) or detection of somatic mutations by ultrasensitive sequencing of a specific panel of genes (CAPP-Seq — cancer personalized profiling by deep sequencing).

IgNGS employs the determination of a tumor clone using a set of universal primers for PCR amplification of immunoglobulin genomic regions with subsequent sequencing of all amplified VDJ rearrangements. Using this method, tumor clones can be detected and monitored using particular Ig gene sequences of the malignant cells. The level of a particular Ig rearrangement to be considered a clonal amplification was arbitrarily set at 5% of all Ig sequences in a given sample.

The CAPP-Seq method is based on analysis of a panel of genes frequently mutated in a particular disease. Exons (and eventually other regions) of genes of interest are custom captured and sequenced by NGS. In contrast to sequencing DNA extracted directly from the tumor, cfDNA sequencing requires much higher coverage of captured regions (in thousands of reads) to enable higher sensitivity and reliability of ctDNA detection. In comparison to IgNGS, CAPP-Seq enables simultaneous monitoring of multiple mutations. The ctDNA concentration for its quantitative analysis is determined from the total concentration of cfDNA (measured after its extraction from plasma). The average proportion of mutated vs. normal reads for detected tumor specific mutations reflects the proportion of ctDNA from total cfDNA. The consensus is to report the ctDNA concentration in human haploid genome equivalents per ml of plasma. The ctDNA concentration in ng/ml of plasma is divided by the amount of DNA present in one human haploid genome (approximately 3.3 pg) [57]. CAPP-Seq also provides information about mutational spectrum in individual patients and eventual clonal evolution but is limited to the analysis of pre-designated sets of genes. Another CAPP-Seq limitation is related to tumor mutation detection sensitivity. The minimal residual disease (MRD) monitoring using CAPP-Seq of cfDNA is limited not only by low input DNA but also by error rate of currently available sequencing technologies. Several methods were suggested to overcome this limitation: molecular barcoding strategies tagging individual DNA molecules to eliminate PCR duplicates, digital in silico elimination of background artifacts, or duplex sequencing (mutations need to be present simultaneously on forward and reverse sequencing reads) [58–60]. Recently, Kurtz et al. introduced an alternative method to duplex sequencing: a phased variant enrichment and detection sequencing (PhasED-seq) [61]. It is based on detection of phased variants (on the same strand of sequenced DNA). It lowers the variant detection sensitivity and improves error discrimination by simultaneous detection of two (or more) variants in one DNA molecule.

## Genetic profile of Hodgkin lymphoma

Defining the genetic background of HL is complicated by the low percentage of HRS cells within the tumor, requiring technically complicated microdissection or sorting. Therefore, relatively fewer studies have been published about HL genetics in comparison to other cancer types. Since HL is not a uniform disease, the genetic profile of somatic mutations also differs between HL subtypes. According to the current WHO histological classification, HL is divided into two subsets: classical HL (cHL), which accounts for approximately 90–95% of all HL cases, and nodular HL with lymphocyte predominance (NLPHL). cHL is further divided into four histological subtypes: nodular-sclerotic (NS), mixed cellularity (MC), lymphocyte predominance, and lymphocyte depletion [62].

Signaling pathways and processes shown to be repeatedly affected by somatic alterations in cHL are NF-κB signaling (*NFKBIA*, *NFKBIE*, *TNFAIP3*, *IKBKB*, *REL*), PI3K-AKT signaling (*ITPKB* and *GNA13*), NOTCH signaling (*SPEN* and *FBXW7*), JAK/STAT signaling (*JAK2*, *SOCS1*, *PTPN1*, *STAT6*), or general regulation of tyrosine kinases signaling (*PTPN2*) and RNAs/proteins nuclear export regulation (*XPO1*) [63–67]. The most frequently altered genes in cHL are *STAT6* (30–80% of cases), *SOCS1* (40–60% of cases), *REL* (50% of cases), *TNFAIP3* (16–40% of cases), *B2M* (16–36% of cases), or *JAK2*, *PDL1*, and *PDL2* (all altered in approximately 30% of cases) [57, 64–69]. Moreover, mutations and/or amplifications in genes like *CIITA*, *PDL1/2*, *PTPN1*, or *B2M* affect the HRS cells interaction with tumor microenvironment causing escape from immune surveillance [65, 70, 71]. Importantly, genetic heterogeneity also exists within cHL subtypes. *STAT6* and *TNFAIP3* mutations are more frequent in the cHL-NS subtype in comparison to other cHL subtypes and more common in Epstein-Barr virus (EBV)-negative cHL as compared to EBV-positive cases [72]. Around 30–40% of cHL cases are EBV positive. The expression of EBV latent membrane protein 1 (LMP-1) leads to NF-κB signaling pathway activation, prevents apoptosis of HRS cells, and results in their long-term survival [73].

Similarly, as in cHL, genes mutated in NLPHL affect multiple critical signaling pathways and cell regulatory circuits: (1) genes involved in the regulation of basic cellular processes such as transcription (*TCEB3*), translation (*EIF3A*), chromosomal stability (*HIST1H3G*), and DNA damage-dependent post-translational modifications of histones (*PARP14*); (2) genes involved in the regulation of inflammatory processes, such as *DUSP1*, *DUSP221*, *NFATC322*, and NF-κB activating kinase *TBK123*; and (3) oncogenes and tumor suppressor genes such as *MYC* and MYC-target genes *MYCT1*, *HHEX24*, and *JUNB25* [74]. The most common alterations in NLPHL are mutations of the *DUSP2*, *SGK1*, *JUNB*, and *SOCS1* genes, all mutated in approximately 50% of cases [64]. Translocations involving the *BCL6* locus (a major lymphoma associated transcriptional repressor) are another typical NLPHL genetic alteration. *BCL6* translocations are almost exclusively present in NLPHL and are very rare in cHL [75–77]. On the other hand, *STAT6* and *JAK2* mutations (very frequent in cHL) were not found in NLPHL. This is surprising since aberrant activation of the JAK/STAT signaling pathway was shown to contribute to the pathogenesis of NLPHL as well. In NLPHL, mutations in the *SOCS1* gene lead to constitutive activation of STAT transcription factors by disruption of SOCS1 functions regulating the activity and degradation of JAK2 kinase [78].

Signaling pathways and cellular processes deregulated in NLPHL and cHL are to a certain degree similar to other lymphoma types; however, they differ in certain specifics. One such example is the overactivation of the NF-κB pathway. In cHL, oncogenic constitutive NF-κB activation results from inactivating mutations of genes encoding negative NF-κB regulators IκBα and A20 (*NFKBIA* and *TNFAIP3*, respectively). Interestingly, these mutations are not responsible for NF-κB dysregulation in NLPHL. Similarly, genomic gains of the *REL* gene (coding for c-Rel, a member of NF-κB family of transcription factors) have been detected in cHL, but they appear to be rare or absent in NLPHL [79]. It is therefore interesting that based on genetic studies, NLPHL appears to be more related to diffuse large B-cell lymphoma (DLBCL), and in particular to the T-cell/histiocyte-rich DLBCL subtype, than is to cHL [80–82].

## Circulating tumor DNA in cHL and its prognostic value

As mentioned above, the genetic studies of cHL are quite difficult due to a low percentage of tumor cells within the total tumor mass. Therefore, detection of tumor-associated mutations using ctDNA is particularly appealing in this tumor type. It was shown that despite the low percentage of the tumor cells in HL tumors, HRS cells release DNA into circulation in relatively high quantities.

Spina et al. analyzed plasma cfDNA samples from 80 patients with newly diagnosed cHL and 32 patients with refractory cHL using a CAPP-Seq approach with a panel of 77 genes. The *STAT6* gene was identified as the most frequently mutated gene in cHL (in 35% of cfDNA samples from newly diagnosed cHL) [72]. In 15 patients, tumor DNA from HRS cells obtained by microdissection from paired biopsies was also available for comparison. In total, 106 mutations were detected from plasma ctDNA, and 96 mutations were detected from tissue biopsy. Mutations detected only through ctDNA analysis (those mutations which were not confirmed from paired biopsy) were not detectable in cfDNA after patients achieved complete remission, which strongly supports the assessment that these mutations were derived from sub-clonal HRS tumor cells located at a different anatomical location than the site of the paired biopsy. This highlights the possible spatial tumor heterogeneity and the advantage of ctDNA in tumor genotyping [72]. Biopsy analyses also confirmed *STAT6* mutations as the most frequent alterations, but with a substantially higher frequency (80%) than was found in cfDNA. Tumor biopsy specific mutations were detectable in cfDNA with 87.5% sensitivity; however, this analysis is limited by a relatively small number of analyzed tumor samples (15). Most importantly, Spina et al. identified exceptional correlation between the decrease of ctDNA load after 2 cycles of chemotherapy and patient prognosis. A 2 logarithm decrease in plasma ctDNA concentration after 2 cycles of ABVD chemotherapy (in comparison to the pre-treatment level) highly correlated with treatment outcome. All patients that did not reach 2 logarithms decrease after 2 cycles of chemotherapy turned out to have a progressive disease [72]. This correlation was much better in the prediction of disease progression when compared to a standard interim PET/CT exam. This strongly suggests that ctDNA could potentially be used as a novel prognostic biomarker in patient risk stratification. An overview of the study by Spina et al. and all other discussed HL ctDNA studies is provided in Table 2 summarizing the most frequently altered genes in individual studies. A study of 96 newly diagnosed childhood HL (Desch et al. 2019, NGS panel of 121 genes) patients confirmed that the initial pretreatment concentration of ctDNA correlates with the total metabolic volume as assessed by PET/CT. Furthermore, the authors observed a similar correlation of ctDNA decrease after initial treatment and prognosis as Spina et al. The ctDNA detectability following initial treatment also correlated with the positivity of early treatment PET/CT result. The most frequently mutated gene in childhood HL was *SOCS1* (80% of cases) [83].

**Table 2:** Overview of NGS based ctDNA studies in Hodgkin lymphoma

Study	Type of study	Analyzed genes	Most frequently mutated genes, % of samples with mutation					
			HRS cells biopsy		ctDNA prior treatment		ctDNA in relapse	
Spina et al. 2018 [72]	ctDNA in 112 cHL patients (80 newly diagnosed and 32 relapsed/refractory), paired biopsies in 15 patients	NGS panel of 77 genes	*STAT6* *ITKPB* *TNFAIP3* *GNA13* *B2M*	80% 53% 35% 27% 27%	*STAT6* *TNFAIP3* *ITPKB* *GNA13* *B2M*	38% 35% 28% 19% 16%	*STAT6* *GNA13* *TNFAIP3* *ITPKB* *ARID1A* *TET2* *BCOR* *SPEN*	28% 28% 25% 19% 19% 16% 16% 16%
Desch et al. 2019 [83]	ctDNA + paired biopsies in 96 newly diagnosed pediatric HL patients	NGS panel of 121 genes	*SOCS1* *IGLL5* *TNFAIP3* *STAT6* *NFKBIE*	60% *	*SOCS1* *IGLL5* *TNFAIP3* *STAT6* *NFKBIE*	83% *	N/A	
Bessi et al. 2018 [68]	ctDNA + paired biopsies in 24 newly diagnosed cHL patients	NGS panel of 6 genes (*B2M, STAT6, XPO1, NFKBIE, PTPN1 TNFAIP3*)	*STAT6* *XPO1* *PTPN1* *TNFAIP3* *B2M* *NFKBIE*	38% 29% 20% 17% 17% 8%	*STAT6* *XPO1* *B2M* *TNFAIP3* *PTPN1* *NFKBIE*	29% 21% 25% 17% 8% 4%	N/A	
Camus et al. 2021 [57]	ctDNA in 60 newly diagnosed cHL patients, 30 paired biopsies	NGS panel of 9 genes (*NFKBIE, ITPKB, PTPN1, TNFAIP3, SOCS1, STAT6, B2M, XPO1, GNA13*)	*SOCS1* *B2M* *TNFAIP3* *STAT6* *ITPKB*	57% 37% 27% 27% 23%	*SOCS1* *B2M* *TNFAIP3* *STAT6* *ITPKB*	48% 30% 32% 18% 22%	N/A	
Alcoceba et al. 2021 [84]	ctDNA in 49 newly diagnosed cHL patients	NGS panel of 42 genes	N/A		*SOCS1* *IGLL5* *TNFAIP3* *GNA13* *STAT6*	28% 26% 23% 23% 21%	N/A	
Sobesky et al. 2021 [85]	ctDNA in 121 newly diagnosed cHL patients	Three different versions of a custom NGS panel **	N/A		*TTN* *SOCS1* *TNFAIP3* *ITPKB* *STAT6*	64% 52% 46% 45% 38%	N/A	
Di Trani et al. 2020 [86]	ctDNA in 20 relapsed/refractory cHL patients	NGS panel of 133 genes	N/A		N/A		*STAT6* *SOCS1* *TNFAIP3* *KMT2D* *ITPKB* *GNA13*	50% 50% 45% 45% 40% 35%
Shi et al. 2020 [87]	ctDNA in 75 relapsed/refractory HL patients	NGS panel of 659 genes	N/A		N/A		*STAT6* *TNFAIP3* *LRP1B* *B2M* *PCLO*	46% 42% 38% 38% 38%

*The frequencies of mutations of the remaining listed genes are between 20 and 30%. Exact frequencies are not provided in the study by Desch at al. **Information about the individual versions of the NGS panel including number and list of analyzed genes was not provided in the study by Sobesky et al.

Bessi et al. used an NSG panel targeting 6 selected genes (*B2M*, *STAT6*, *XPO1*, *NFKBIE*, *PTPN1*, and *TNFAIP3*) to compare the mutational spectrum of matched tissue biopsies and plasma collected before treatment in 24 cHL patients. Mutation in at least one of the analyzed genes was detectable in 13/24 biopsies (54.2%) and 11/23 plasma samples (47.8%). In 7/23 cases (30.4%), similar somatic mutations were detected in cfDNA as in the matched tissue biopsy. The most frequently mutated genes included *STAT6* and *XPO1*. *STAT6* gene mutations were detected in 9 tumor biopsies (37.5%), but in only 7 matched cfDNA samples (29%). The *XPO1* mutations were detected in 7 tissue biopsies (29%) and in 5 matched cfDNA samples (20.8%). Interestingly, two patients had detectable *XPO1* mutation in cfDNA, but not in paired biopsy [68]. The failure to detect mutations in cfDNA that were present in tissue biopsies highlights one of the challenges of ctDNA analyses: the sensitivity of mutations detection.

Despite a relatively small group of analyzed patients, the above-mentioned studies showed promising results and identified mutations of *STAT6*, *XPO1*, or *SOCS1* genes as possible candidates for MRD monitoring. *XPO1* gene mutations in cfDNA for MRD monitoring were previously studied by Camus et al. [63]. They analyzed tissue biopsies of 94 cHL patients by ddPCR at the time of initial diagnosis with 50 matched cfDNA samples. *XPO1* mutations were found in tissue samples of 22 patients (24.2%). In 5 patients, *XPO1* mutation was detectable only in the tumor biopsy, but 8 patients had *XPO1* mutation detectable only by cfDNA (29% of all *XPO1* mutations). These results support the importance of a joint tumor and ctDNA analysis for best mutation detection sensitivity. Additionally, in 28 patients, the variant allele frequency (VAF) of *XPO1* mutation in plasma was analyzed at the end of the treatment. The VAF decreased in most patient samples (*n* = 20) and became undetectable in 16. Patients with a detectable *XPO1* mutation at the end of treatment displayed a trend toward shorter 2y-progression free survival (PFS), as compared to patients with undetectable mutation (2y-PFS = 57.1%, CI 95%:30.1–100% versus 2y-PFS = 90.5%, CI 95%:78.8–100%, respectively, *p* = 0.0601). Overall, disease relapse occurred in 26 out of the 94 analyzed patients (28%). However, disease relapse occurred in 4 out of 7 patients with *XPO1* mutation detectable at the end of the treatment (57%) which suggests a superior sensitivity of liquid biopsy over PET/CT for relapse prediction as only one out of the 7 *XPO1* positive patients had a positive PET/CT at the end of the treatment [63].

To confirm the previous results and to search for more potential biomarkers, Camus et al. prospectively analyzed cfDNA samples of 60 cHL patients (and 30 matched biopsies) using an NGS panel of 9 genes: *NFKBIE*, *TNFAIP3*, *STAT6*, *PTPN1*, *B2M*, *XPO1*, *ITPKB*, *GNA13*, and *SOCS1* [57]*.* Overall, at least one mutation was detected in 42 (70%) patients, in 41/60 (68.8%) of cfDNA samples, and in 21/30 (71%) of available biopsies. The most frequently mutated gene was *SOCS1* (51.7% of patients), followed by *B2M* (33.3%), *TNFAIP3* (31.7%), *ITPKB* (23.3%), and *STAT6* (23.3%). This study reported a match in mutation spectrum between biopsy and cfDNA samples in 25 out of 30 samples. Plasma ctDNA after 2 cycles of chemotherapy (C2) was analyzed in 41 patients with at least one mutation detected at diagnosis. Interestingly, no somatic variant was detected in any of the analyzed patients following the initial treatment, which might have been affected by a study specific detection sensitivity threshold. Possible approaches to improve the sensitivity of MRD detection are discussed above. In lymphoma, Alcoceba et al. analyzed 49 newly diagnosed cHL patients using an NSG panel covering 42 genes and implemented unique molecular identifiers (UMIs) into the sequencing workflow [84]. The most frequently mutated genes were *SOCS1* (28%), *IGLL5* (26%), *TNFAIP3* (23%), *GNA13* (23%), and *STAT6* (21%). Although the use of UMIs increased stringency of mutation calling and decreased false positivity, it resulted in lower frequencies of detected mutations in comparison to other studies. ctDNA dynamics following initial treatment was not evaluated in this study. The prognostic value of ctDNA in HL was most recently confirmed by Sobesky et al. [85]. The authors analyzed ctDNA in 121 newly diagnosed cHL patients using three different versions of a custom NGS panel. The most frequently mutated genes were *TTN* (64%), *SOCS1* (52.3%), *TNFAIP3* (45.9%), *ITPKB* (45%), and *STAT6* (37.8%). Importantly, all patients with undetectable ctDNA after 2 cycles of chemotherapy achieved PET negativity at the end of treatment. Conversely, patients with residual MRD positivity after 2 cycles of chemotherapy were PET positive at the end of treatment. Moreover, ctDNA assessment after only 1 week of treatment was highly predictive of early PET response after first 2 cycles of chemotherapy. The authors identified 6 different mutational signatures reflecting different mechanisms of mutations acquisition. Mutations in certain genes were associated with unfavorable phenotype (e.g., *TP53* or *NOTCH1* mutations) or better treatment response (e.g., *STAT6* amplification, *BRCA1* loss, or *EGMT* loss). Analysis of tumor clonality identified several genes with different mutation frequencies between the main clones and subclones, however, did not identify any specific order of mutations occurrence during lymphomagenesis.

HL, as in a majority of tumors, consists of oligoclonal populations of malignant cells with clonal selection and/or clonal evolution frequently occurring during the course of the disease. This is one of the major factors responsible for acquired chemoresistance and disease relapse [72, 88]. In the above-mentioned study by Spina et al., clonal evolution between pre-treatment and relapsed samples was identified in all tested cases [72]. The *STAT6* gene mutations (and other most frequent mutations) were already detectable in the so-called ancestral clones forming the initial tumor at diagnosis. Since these mutations persisted intact throughout the course of the disease, they are not the primary cause of acquired chemoresistance or relapse [72]. To assess the possible clonal evolution, Di Trani et al. analyzed ctDNA of 20 relapsed/refractory (r/r) cHL patients treated with checkpoint inhibitors by CAPP-Seq of 133 lymphoma associated genes [86, 89]. The most frequently mutated genes at the time of the relapse were *STAT6* (50%), *SOCS1* (50%), *TNFAIP3* (45%), *KMT2D* (45%), *ITPKB* (40%), and *GNA13* (35%). Interestingly, two patterns of evolution during the treatment were detected: “clonal reshaping,” where original mutations disappeared and new mutations occurred; and “clonal persistence,” where original mutations stayed present during the treatment. Clonal reshaping was associated with sensitivity and clonal persistence with resistance to treatment. This pattern was also correlated with PET/CT results. Median PFS between clonal reshaping and clonal persistence groups was 39.5 vs. 9.5 months, respectively (*P* < 0.003). In another study, Shi et al. analyzed the cfDNA samples of 75 r/r cHL patients using a set of 659 genes to identify mutation patterns at relapse and possible association of specific mutations with PFS and eventual resistance to anti PD-1 treatment (sintilimab) [87]. The most frequently mutated genes included *STAT6* and *TNFAIP3* (both mutated in approximately 35% of cases), *SOCS1*, *B2M*, *LRP1B*, *PCLO* (all mutated in approximately 25% of cases), or *MLL2*, *GNA13*, and *TP53* (all mutated in approximately 20% of cases). The spectrum and frequencies of identified mutations were similar to studies analyzing treatment naïve tumors/ctDNA with the exception of *PCLO* and *LRP1B* genes (uniquely mutated in this study). However, there are no data available at diagnosis to distinguish if these mutations were the result of therapy-driven clonal evolution. Interestingly, *CHD8* gene mutations were detected only in patients with PFS ≥ 12 months (23%, 6 out of 26 patients) in comparison to patients with PFS < 12 months (none of 20 patients), suggesting it as a possible positive biomarker of anti PD-1 treatment of r/r cHL. A ctDNA VAF decrease of more than 40% correlated with the response to anti PD-1 antibody treatment and mirrored radiographic response during therapy but could not predict PFS. This corresponds to a similar observation of ctDNA load decrease predicting response to cHL first line treatment in Spina et al. [72]. From 12 patients with acquired resistance to sintilimab, 9 had positive detection of new mutations in their plasma samples: *B2M* (2/12), *TNFRSF14* (2/12), *KDM2B* (2/12), *S1PR2* (1/12), *NFKB2* (1/12), and *RELN* (1/12). A relatively wide spectrum of post treatment mutations suggests that multiple mechanisms/genes might be associated with anti-PD-1 treatment resistance development, but much larger analysis needs to be performed.

One of the main disadvantages of a CAPP-Seq approach that focuses on a pre-set number of genes is the limited ability to detect novel mutations that develop during the course of the disease. Studies using much larger panels and/or whole exome sequencing would therefore be needed to map the mutational pattern and heterogeneity following initial treatment and relapse.

## Conclusion

Taken together, the above-mentioned studies consistently report relatively high correlation of mutations detected from ctDNA vs. from HL tumor cells. In some instances, certain mutations were detectable only from ctDNA, which highlights its potential capacity to better report on the overall genotype and clonality of tumors regardless of anatomical site. Although it seems that pretreatment ctDNA levels correlate well with the tumor burden [90], HL ctDNA studies demonstrated that following the initial treatment, PET/CT imaging positivity or negativity does not always correlate with the magnitude of plasma ctDNA decrease and further showed that this initial ctDNA decrease is highly predictive of the course of the disease. This discrepancy between standardized uptake value of contrast agent on PET/CT and the decrease in plasma ctDNA concentration post initial treatment could potentially be explained by the fact that the maximum standardized uptake value corresponds to the metabolic activity of the reactive microenvironment, whereas ctDNA reflects the level of tumor burden.

The fast-evolving non-invasive ctDNA detection technology might be suitable to monitor MRD during and after treatment, to monitor the clonal evolution of the disease, and to predict the relapse or chemoresistance. Therefore, it might well complement PET/CT exams in the management of HL patients. On the other hand, genotyping and monitoring of the mutation load using ctDNA to assess the patient’s risk profile are not yet at the stage to be routinely used for HL patient management and is applied only at an experimental level. Standardizations of the protocols at all stages of tumor mutation detection, defining the panels of the most relevant genes, performing prospective studies of ctDNA predictive value, and assessment of its usability in routine testing are necessary before analysis of ctDNA could be moved to clinical use.
